# Hidden Curriculum in Medical Residency Programs: A Scoping Review

**DOI:** 10.30476/JAMP.2021.92478.1486

**Published:** 2022-04

**Authors:** GHADIR POURBAIRAMIAN, SHOALEH BIGDELI, SEYED KAMRAN SOLTANI ARABSHAHI, NIKOO YAMANI, ZOHREH SOHRABI, FAZLOLLAH AHMADI, JOHN SANDARS

**Affiliations:** 1 Center for Educational Research in Medical Sciences (CERMS), Department of Medical Education, School of Medicine, Iran University of Medical Sciences, Tehran, Iran; 2 Medical Education Research Center, Isfahan University of Medical Sciences, Isfahan, Iran; 3 Nursing Department, Faculty of Medical Sciences, Tarbiat Modares University, Tehran, Iran; 4 Edge Hill University Medical School, Edge Hill University, Ormskirk, UK

**Keywords:** Hidden curriculum, Medical education, Curriculum, Education

## Abstract

**Introduction:**

Hidden curriculum is important in medical education and has numerous, long-lasting effects on medical residency. The present scoping review seeks to investigate, identify, and plot the main concepts relating to hidden curriculum and its dimensions, domains, impacts and factors in medical residency courses based on the main references and evidence.

**Methods:**

Scoping review methodology was used to guide a search of electronic databases for relevant papers. Of the 394 abstracts initially identified, after screening of both abstracts and full-text papers, 43 studies were selected for inclusion in this review. Following abstraction of key information from each study, a content analysis was undertaken.

**Results:**

Eleven themes were identified from the content analysis: 1) Organizational Issues (13.77%), 2) Socio-cultural Issues (10.5%), 3) Professional Issues (13.41%), 4) Communicational Factors (8.7%), 5) Educational Issues (22.83%), 6) Resident Personal Characteristics (21.01%), and 7) Resident Educational Characteristics (9.78%). Among the extracted effective hidden curriculum factors, the role modeling had the highest frequency and was emphasized in the studies.

**Conclusions:**

Although this study explained and identified the components, elements and also the preparation of the initial format of the hidden curriculum framework of the medical residency program, its results can reduce the negative effects of the hidden curriculum on it. More extensive and in-depth studies with different qualitative methods or mixed methods related to the hidden curriculum in different contexts and disciplines of medical residency are recommended to define characteristics of a constructive hidden curriculum of medical residency programs.

## Introduction

The curriculum has a major influence on the educational outcomes and experiences of learners ( 1 ). There have been several different classifications of the curriculum based on different criteria. Eisner highlights the important distinction between the explicit and hidden curriculum ( 2 ). The explicit curriculum refers to the planned and expected educational outcomes and experiences of learners, but there are other inevitable factors that influence their experiences, transfer of ideas, attitudes, values, and behaviors. Such experiences that are not explicitly present in the predefined curriculum is referred to as hidden curriculum ( 3 ). 

Historically, the concept of the "hidden curriculum" dates back to Philip Jackson in the book “Life in Classrooms” ( 4 , 5 ). However, many other educationalists and researchers, such as Everly, Dewey, Apple, Giroux and Eisner, have also discussed the dimensions and nature of a hidden curriculum ( 6 ). The term hidden curriculum refers to the unwritten, unofficial, and often unintended values, views, and lessons that are learned in educational environments ( 7 ). Bloom suggested that a hidden curriculum is often more effective and efficient than more explicit curriculum aspects ( 8 ), especially about the expected values, beliefs, and behaviors ( 9 ). 

 The hidden curriculum in medical sciences education has been extensively discussed by Hafferty, who defined it as a set of influences at the organizational and cultural levels, including organizational policies, evaluation activities, resource allocation decisions, and intra-organizational policies ( 10 ). The hidden curriculum has a strong potential in the clinical environment to influence the current and future clinical and professional performance of both learners and their educators since it conveys the important ethical culture, norms, and rules about appropriate feelings and behavior ( 11 ). 

Residency or postgraduate training is specifically a stage of graduate medical education. It refers to a qualified physician, podiatrist (one who holds the degree of MD, DO, DPM, MBBS, MBChB) who practices medicine, usually in a hospital or clinic, under the direct or indirect supervision of a senior medical clinician registered in that specialty such as an attending physician or consultant. Residency is one of the most important educational courses related to health sciences. It aims at training qualified and committed physicians to contribute to theoretical and practical skills, medical education, healthcare expansion, treatment, and research and to extend medical sciences. While practicing independently is possible, the vast majority of physicians choose to pursue a residency for further training. Residency can range from an additional two years of education to an additional seven years of training, depending on the specialty. A medical residency takes place in a hospital or clinic and provides in-depth training within a specific medical specialty. Doctors develop skills in laboratory work, medical procedures, patient care, quality control, and self-care. Residency is intensive and difficult, and doctors work long hours as they put their classroom and clinical experience into practice ( 12 - 18 ).

Residents are an essential part of the medical workforce in many countries and they are often asked to train medical students due to the lack of teaching staff in hospitals. Thus, residents may play a three-pronged role: In the day-to-day work schedule of hospitals, residents are both inclusive and responsible for caring of patients, and are assigned a portion of medical students' education. Therefore, it is very important to balance teaching, learning and health care provided by them ( 19 , 20 ).

The learning and teaching process in the residency program is very dynamic and involves a complex interaction between different factors. For example, workload and work environment, formal training activities and close supervision and feedback from faculty, etc. all affect learning in the residency program. The Accreditation Council for Graduate Medical Education (ACGME) in the United States has determined six learning outcomes for postgraduate medical education: patient care, medical knowledge, interpersonal communication skills, professionalism, practice-based learning, and system-based practice ( 21 ). The most notable feature of medical residency is in-service training, in which teaching is integrated with practical scenarios, thus creating a model for physicians' ideological, moral, and occupational identities. The professional competence expected at the end of the residency is beyond technical knowledge. It also includes skills and attitudes that demonstrate effective team skills, leadership, communication skills, empathy, self-control and metacognition. Medical students also choose residency students as their role models ( 22 ).

 Hidden curriculum strongly affects the norms and values of residents. Research has shown that more than half of residency graduates claimed that they found their expectations of professional attitude and behavior to be different from the behaviors of their clinical teachers ( 19 ). The hidden curriculum not only shapes the learning of residents but also conceptually changes the idea of how a resident could perform in a complex healthcare system. Physicians obtain new experiences that may create a gap between reality and actions. The messages of hidden curriculum may help physicians reduce the experienced anomalies between the ideal performance and current performance. In fact, the outcomes of hidden curriculum compete with researches published in journals and rules published in guidelines. The awareness of the position of hidden curriculum provides an opportunity to discover factors that influence not only the behavior but also the therapeutic responses and decisions of physicians ( 10 , 20 ). This suggests a gap between explicit and hidden curriculum. 

Residency is a time-consuming stage, and residents are obsessed in the hospital culture and thus further influenced by hidden curriculum. Negative effects of hidden curriculum would replace professional values such as altruism, trustworthiness, and sympathy with opportunism, pessimism, and indifference ( 21 ). A number of studies reported increased post-residency unprofessional behaviors. As moral behaviors and attitudes are often dependent on education circumstances, residents are more likely to exert unprofessional behaviors in stressful workplaces under the dominance of negative hidden curriculum effects ( 22 , 23 ). 

Such environments may even impact the personal health of residents. Burnout is defined as the syndrome of abreaction and adaptive indifference after long exposure to job stress. It is estimated that 50-76% of residents experience burnout. This reduces sympathy, weakens caregiving, and increases medical errors, leading to increased unprofessional behaviors of residency students ( 24 - 26 ). 

Billings, et al. (2011) investigated the influences of hidden curriculum on internal residents at medical schools in Washington D.C. and Los Angeles and revealed that residents were treated unprofessionally ( 19 ). In a qualitative case-study work, Van-Don, et al. (2013) identified hidden curriculum in radiology residency within five radiology centers at Canadian hospitals. The results suggested that an isolation-oriented hidden curriculum governed four of the five radiology centers, and radiology residents were treated as independent operators ( 27 ). Esteghamati, et al. (2016) qualitatively studied the learning of internal and surgery residency groups in the educational hospitals of Tehran University of Medical Sciences, Iran, and divided the influential factors into three groups: 1) hidden curriculum, 2) learning source, and 3) learning conditions. The hidden curriculum was divided into the clinical culture and clinical priority subgroups. Residents learn to be professional under clinical conditions and individual culture and realize how they can improve their skills of interaction with patients and colleagues ( 28 ). Gupta, et al. (2016) qualitatively evaluated the hidden curriculum of ethics in psychiatry at the higher education level in Canada. They demonstrated that the effective education of ethics in psychiatry curriculum at the higher education level required hidden curriculum direction. They also suggested that not only professional efforts but also a hidden curriculum mechanisms were necessary ( 29 ). Yamani, et al. (2010) and Shakour, et al. (2018) studied professionalization in medical education and found that positive and negative experiences in professional learning implied the contributions of hidden curriculum to clinical education ( 30 , 31 ). 

A review of the expected and predicted contributions and outcomes of residency suggests that some of these contributions and outcomes are educated through hidden curriculum based on the environment and culture of residency sections rather than through explicit curriculum ( 32 ). 

Considering the mentioned issues about the importance and the lasting effects of the hidden curriculum in medical residency courses, how can we identify its positive and negative effects without a correct, scientific and evidence-based understanding of the dimensions and components of the hidden curriculum and take appropriate strategies to deal with its negative and strengthen its positive effects? In addition, considering the importance of medical residency courses in various aspects, including the breadth and number of students, the multiplicity of tasks and roles, training of medical students in internships and externship, the multiplicity of follows branching out from them and that most of these students' training courses are done in clinical settings, the importance of carefully examining and identifying the dimensions and components of the hidden curriculum is doubled.

By recognizing the dimensions and components of the hidden curriculum in residency courses presented in few studies and evidence in this field, effective steps can be taken to identify its unknown effects and improve this dimension of the curriculum. Also, considering very few review studies on the hidden curriculum in the medical residency course, the necessity of designing and conducting an in-depth review study in the form of a scoping review to accurately identify the components and dimensions of the hidden curriculum becomes clearer.

## Methods

The present scoping review is based on Arksey and O'Malley’s six-stage methodology ( 33 ) developed by Levac, et al. ( 34 ). The sixth stage is consultation and is optional. The stages are described below.

### 1. Developing the research question

The present study focused to answer the question “What are the impacts, domains, factors, and components of the hidden curriculum in residency programs?” Although the authors initially intended to focus on internal residency programs, they decided to extend the scope of the study to all residency branches as little literature was available on hidden curriculum in internal residency programs.

### 2. Identifying relevant studies

This stage includes identification of relevant studies and developing a decision-making scheme on the search space, terms, references, time period, and language. The present work focused on the research question using appropriate keywords and searched for hidden curriculum aspects, factors, impacts and domains in residency. To perform a comprehensive search, in the present work all the online databases were searched on the hidden curriculum. Thus, a total of 11 English databases including PubMed, Scopus, Science Direct, Web of Science, ERIC, CINAHL, PsycINFO, EBSCO, Cochrane, ProQuest and Google Scholar, were searched. The search strategy was developed with a librarian having expertise in data searching and working in the university library of the key researchers of this study. The search strategies of the databases were based on certain keywords, Boolean operators, and the specific coordinates of advanced search as follows: PubMed: (("hidden curriculum"[Title/Abstract] OR "Covert Curriculum" [Title/Abstract] OR "Latent Curriculum" [Title/Abstract] OR "Invisible Curriculum" [Title/Abstract]) AND (residency [Title/Abstract] OR resident) AND (internal medicine [Title/Abstract]) AND (y_10[Filter]) AND ((y_10[Filter]) AND (clinicaltrial[Filter] OR journalarticle[Filter] OR meta-analysis[Filter] OR preprint[Filter] OR randomizedcontrolledtrial[Filter] AND (english[Filter])). Scoping reviews are performed to plot the main concepts of a study and the main references and evidence, and can be conducted as independent work, particularly when dealing with a context that is complicated or has never been addressed. Thus, studies published during 2011-2020 were selected to extract evidence( 33 ). Also, it should be noted that at the time of this research, it was not possible to access foreign grey literature in English language (university dissertations, government documents, organizational documents and others), so this research does not include review of grey literature.

### 3. Selecting studies

**Figure 1:**
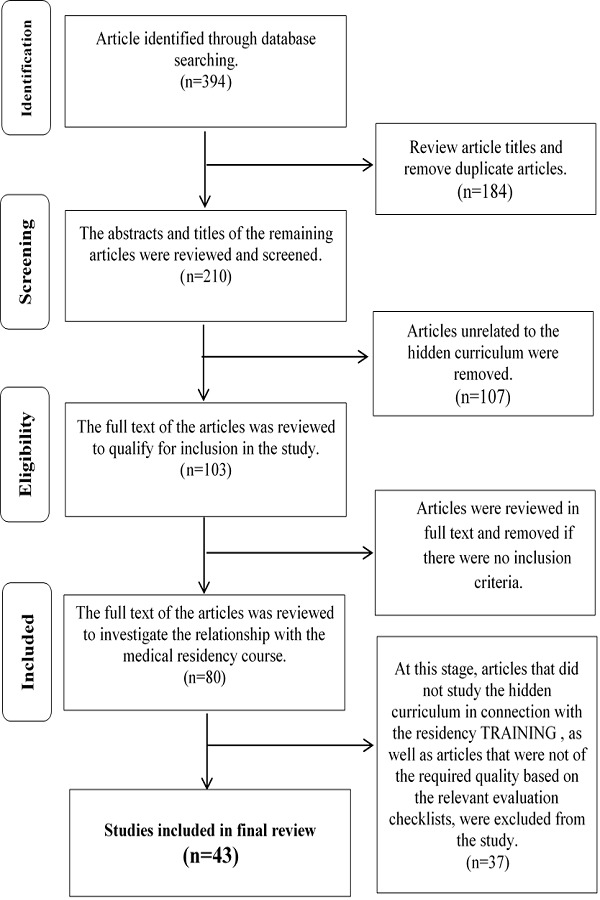
Flow diagram of the literature search and study selection process

In this stage, studies were selected, as shown in Figure 1, based on PRISMA, MERSQI, COREQ and MMAT Protocols, respectively for Review, survey, qualitative and mixed methods studies. Invalid and yellow articles have been excluded from the study based on searches in valid databases, which are presented in Part 2 of the Methodology section, as well as the use of the above appraisal protocols. The databases were searched based on the search strategies. The results of the online databases were introduced to EndNote – a total of 394 studies – so that repeated studies could be excluded. Therefore, 184 of the 394 studies were excluded. The remaining 210 studies were delivered to two members of the research team to independently investigate the titles and abstracts. Then, 170 studies with no relevance to hidden curriculum were excluded, and the remaining 103 studies were subjected to the inclusion criteria:

-Explicit investigation of hidden curriculum,
-Investigating hidden curriculum in medical and health contexts,
-Written in English, and
-Not an essay or commentary.

23 of the studies did not meet the inclusion criteria and were excluded, and the remaining 80 studies discussing the hidden curriculum in residency programs were reviewed. 37 of the 80 studies did not explicitly and clearly discuss hidden curriculum and thus were excluded. Finally, a total of 43 studies were included in the review. 

### 4. Charting the data

A charting data form was utilized to extract data from the studies. To extract process-based text data, the narrative review method was adopted as a descriptive-analytical approach ( 33 ). Also, a charting data form was developed by the authors to extract data from the studies. Two of the authors independently extract data relating to the scoping review questions and objectives. The extracted data were compared to ensure the reliability of the adopted approach. The researchers discussed and resolved any lack of consensus if there was any. 

### 5. Collating, summarizing, and reporting results

In this stage, the results were represented by a thematic structure. To present and conclude the results, the present work employed several methods based on multi-dimensional data from the searched online databases. Also, the authors attempted to maintain clarity and stability in the results. 

### Ethical Consideration

This article is the product of the first phase of the PhD thesis of the first author, entitled “Components, elements and dimensions of Hidden Curriculum of Internal Medicine Residency Program and Developing a Conceptual Framework, Iran University of Medical Sciences” in the field of medical education at Iran University of Medical Sciences (IUMS), which has been approved and financially supported by the research committee of this university with the research number of 98-4-4-16588.

This study has been approved, by the ethics committee of School of Medicine, Iran University of Medical Sciences (IUMS) with the ethical code of IR.IUMS.FMD.REC.1398.514.

## Results

### Brief review of the studies

**Figure 2:**
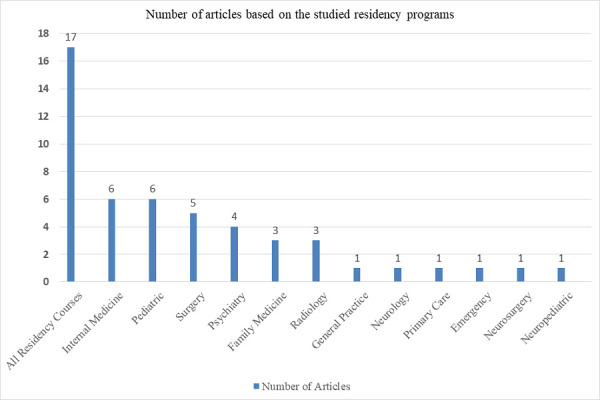
Graph of the studied residency programs

As mentioned, a total of 43 studies were reviewed, of which 17 studies ( 35 - 51 ) examined the hidden curriculum of residency programs in a general context, 6 studies ( 19 , 28 , 52 - 55 ) evaluated the hidden curriculum in internal medicine, 6 studies addressed pediatrics ( 52 , 55 - 59 ), 5 studies incorporated surgery ( 28 , 53 , 60 - 62 ), 4 studies ( 29 , 63 - 65 ) discussed psychiatry, 3 studies ( 52 , 66 , 67 ) investigated family medicine, and 3 studies ( 27 , 68 - 69 ) evaluated radiology residency programs. Also, each of neurology ( 70 ), general practice ( 71 ), primary care ( 72 ), emergency ( 54 ), neurosurgery ( 62 ), and neuropediatric ( 73 ) residency programs had been discussed in a single study. Figure 2 depicts these results.

**Figure 3:**
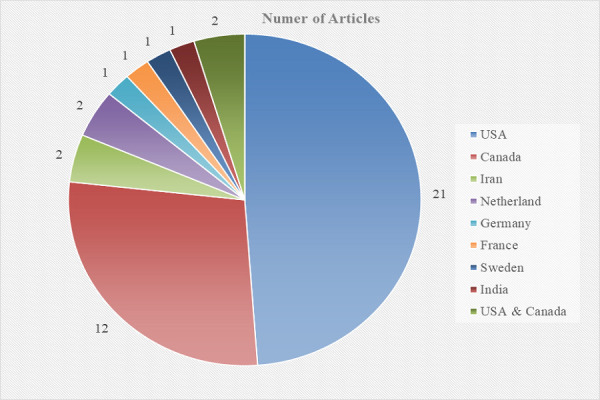
Pie diagram of the distribution of the studies by country

Furthermore, among the 43 studies, 21 studies ( 19 , 35 , 36 , 38 , 41 , 42 , 44 , 46 , 48 , 50 , 52 , 57 , 59 , 61 , 62 , 64 , 65 , 68 , 69 , 72 , 73 ) were conducted in the United States, 12 studies ( 27 , 29 , 39 , 40 , 51 , 54 , 55 , 56 , 58 , 63 , 67 , 70 ) were performed in Canada, two studies ( 28 , 43 ) in Iran, and two studies ( 45 , 53 ) in the Netherlands. Also, a single study had been conducted in Germany ( 37 ), France ( 71 ), Sweden ( 66 ), and India ( 47 ). Two joint studies ( 49 , 60 ) were conducted in Canada and the United States. Figure 3 displays these data. 

According to Table 2, 7 out of the 43 studies adopted mixed methodologies, 4 studies were reviews, 5 studies were quantitative based on questionnaires, and 27 studies adopted qualitative approaches. Several instruments were employed to measure the quality of the studies, as reported in Table 1. Also, Table 2 provides the frequencies of the studies in different years. As can be seen, most studies were conducted in 2019 (8 studies).

**Table 1:** Frequency of the articles by year of publication

Publication Year	Number of Articles
2020	6
2019	8
2018	7
2017	7
2016	4
2015	5
2014	1
2013	2
2012	-
2011	3
Total of Articles	43

**Table 2:** Distribution of articles and the evaluation tools used according to the type of study

Study type	Number of Articles	Relevant evaluation tools
Mixed Methods Study	7	MMAT
Review Study	4	PRISMA
Survey (Questionnaire)	5	MERSQI3
Qualitative Study	27	COREQ4
Total of Articles	43	

MMAT: The Mixed Methods Appraisal Tool; PRISMA: Preferred Reporting Items for Systematic Reviews and Meta-Analyses; MERSQI: The Medical Education Research Study Quality Instrument; COREQ: Consolidated criteria for reporting qualitative studies

### Content analysis and extracting codes, themes, and categories based on the study objectives

The 43 retrieved studies were entirely reviewed and analyzed by two of the authors using qualitative content analysis in order to extract the factors, impacts, components, and domains of the hidden curriculum in residency programs. Qualitative content analysis as an essential method in qualitative research is used for in-depth data investigation of scientific works. It indicates an in-depth analysis of relationships between variables and the relationship network. The extracted codes discussed, and the researchers reached consensus on the content analysis. Table 3 provides the content analysis results and the frequencies of the extracted codes.

**Table 3:** Table of frequency of codes extracted from articles

N.	Code	Frequency	N.	Code	Frequency	N.	Code	Frequency
1	Role Modeling	21	20	Humanity	4	39	Teachers Behaviors	2
2	Professionalism	17	21	Mental Injuries and Discrimination	4	40	Organization Policy	2
3	Organizational Culture	16	22	Priority of Treatment to Education	4	41	Social Structure	2
4	Culture	13	23	teacher-resident Interaction	3	42	Self-directed Learning	2
5	Interprofessional Collaboration and Interaction	13	24	Activity of Residents	3	43	Self-worth	2
6	Resident Behaviors and Inhabits	13	25	Responsibility of Residents	3	44	Self-care	2
7	Values	10	26	Patient-centered Approach	3	45	Clinical Decision Making	2
8	Leadership and management	9	27	Patient- resident Interaction	3	46	Electives and Choices	2
9	Unplanned Teaching and Learning	9	28	Communication Skills	3	47	Welfare Programs	2
10	Empathy	8	29	Rules	3	48	Resiliency	2
11	Professional Identity	8	30	Integrity	3	49	Resident Experiences	2
12	Hierarchy	8	31	Context Factors	3	50	Religious and Spiritual	2
13	Productivity and Self-efficacy	8	32	Professional Culture	3	51	Socio-Economic Backgrounds	2
14	Organizational Structure	7	33	Work-life Balance	3	52	Uncertainty	2
15	Attitudes	6	34	Authority	3	53	Utilization of Social Media	2
16	Mentoring and Supervision	6	35	Autonomy	3	54	Business Education	2
17	Clinical Practice	5	36	Norms	3	55	Evaluation	1
18	Feedback	4	37	Beliefs	2	Total of Codes		276
19	Reflection and Self-reflection	4	38	Ethical Issues	2			

**Figure 4:**
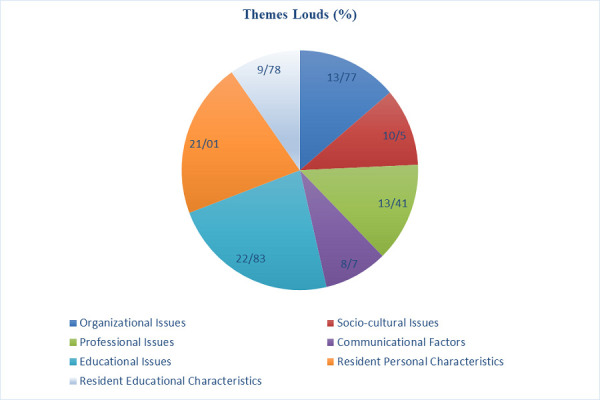
Pie chart of the main themes obtained from the retrieved articles along with their weighted load by percentage

**Figure 5:**
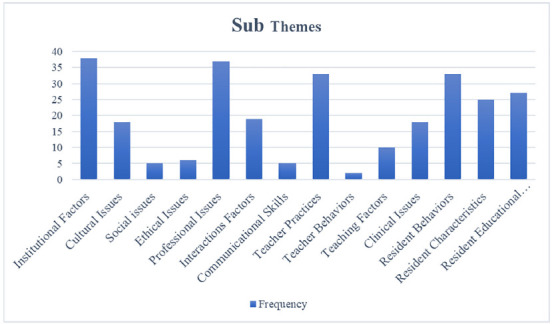
Bar chart of sub-themes resulting from article content analysis

Once the codes had been extracted, the researchers divided the overlapping and associated codes into subthemes. As a result, a total of fourteen subthemes were obtained. Then, the subthemes were further investigated, driving seven themes. Table 4 reports the themes and subthemes along with their codes. Also, Table 5 represents the themes and subthemes along with their weights and their numbers of codes. These data are depicted in the forms of a circular diagram and bar charts in Figures 4 and 5. As illustrated, educational factors had the highest weight (i.e., 22.83% of the total extracted codes).

**Table 4:** Codes, sub-themes and main themes obtained from articles

Codes (Frequency)	Sub Themes (N. of Codes)	Themes (N. of Codes)
Organizational Culture (16)	Institutional Factors (38)	Organizational Issues (38)
Hierarchy (8)		
Organizational Structure (7)		
Organization Policy (2)		
Rules (3)		
Welfare Programs (2)		
Culture (13)	Cultural Issues (18)	Socio-cultural Issues (26)
Context Factors (3)		
Religious and Spiritual (2)		
Social Structure (2)	Social Issues (5)	
Norms (3)		
Mental Injuries and Discrimination (4)	Ethical Issues (6)	
Ethical Issues (2)		
Professionalism (17)	Professional Issues (37)	Professional Issues (37)
Leadership and management (9)		
Professional Identity (8)		
Professional Culture (3)		
Interprofessional Collaboration and Interaction (13)	Interactional Factors (19)	Communicational Factors (24)
Teacher-resident Interaction (3)		
Patient- resident Interaction (3)		
Communication Skills (3)	Communicational Skills (5)	
Utilization of Social Media (2)		
Role Modeling (21)	Teacher Practices (33)	Educational Issues (63)
Mentoring and Supervision (6)		
Feedback (4)		
Business Education (2)		
Teachers Behaviors (2)	Teachers Behaviors (2)	
Evaluation (1)	Teaching Factors (10)	
Unplanned Teaching and Learning (9)		
Clinical Decision Making (2)	Clinical Issues (18)	
Electives and Choices (2)		
Clinical Practice (5)		
Patient-centered Approach (3)		
Priority of Treatment to Education (4)		
Uncertainty (2)		
Resident Behaviors and Inhabits (13)	Resident Behaviors (33)	Resident Personal Characteristics (58)
Values (10)		
Attitudes (6)		
Beliefs (2)		
Socio-Economic Backgrounds (2)		
Resiliency (2)	Resident Characteristics (25)	
Self-worth (2)		
Integrity (3)		
Humanity (4)		
Authority (3)		
Autonomy (3)		
Empathy (8)		
Reflection and Self-reflection (4)	Resident Personal Characteristics (27)	Resident Personal Characteristics (27)
Self-directed Learning (2)		
Resident Experiences (2)		
Activity of Residents (3)		
Productivity and Self-efficacy (8)		
Work-life Balance (3)		
Self-care (2)		
Responsibility of Residents (3)		

**Table 5:** Sub-themes and main themes obtained from articles and their weight

N.	Sub Themes	Frequency	Load (%)	N.	Themes	Frequency	Load (%)
1	Institutional Factors	38	13.77	1	Organizational Issues	38	13.77
2	Cultural Issues	18	6.52	2	Socio-cultural Issues	29	10.5
3	Social issues	5	1.81				
4	Ethical Issues	6	2.17				
5	Professional Issues	37	13.41	3	Professional Issues	37	13.41
6	Interactional Factors	19	6.88	4	Communication Factors	24	8.7
7	Communication Skills	5	1.81				
8	Teacher Practices	33	11.96	5	Educational Issues	63	22.83
9	Teacher Behaviors	2	0.73				
10	Teaching Factors	10	3.62				
11	Clinical Issues	18	6.52				
12	Resident Behaviors	33	11.96	6	Resident Personal Characteristics	58	12.01
13	Resident Characteristics	25	9.06				
14	Resident Educational Characteristics	27	9.78	7	Resident Educational Characteristics	27	9.78
Total		276	100	Total		276	100

## Discussion

### Main findings 

The present study sought to identify the domains, impacts, factors, and components of the hidden curriculum in residency programs and to propose an initial conceptual framework. After the qualitative content analysis of the selected studies, a total of 55 codes with different frequencies were obtained and divided into seven themes and fourteen subthemes. The themes included 1) organizational factors, 2) sociocultural factors, 3) professional factors, 4) communication factors, 5) educational factors, 6) personal characteristics (of residents), and 7) educational characteristics (of residents). As they had the highest numbers of codes, educational factors and personal characteristics of residents obtained the highest weights and importance, while communication factors and educational characteristics of residents had the lowest weights. 

According to the data, many factors are involved in the hidden curriculum of residency programs. Apart from their multiplicity, these factors cover a wide range of areas, including sociocultural factors, personal characteristics of residents, professional factors, educational factors, and organizational factors. Among the extracted effective hidden curriculum factors, the role modeling had the highest frequency and was emphasized in the studies. The effects of the role modeling of teachers, hospital specialists, and experienced residents on inexperienced residents were significantly emphasized. Culture (particularly organizational culture), interactions, and inter-professional cooperation were also frequently mentioned as crucial hidden curriculum factors in the reviewed studies. As with other health disciplines, organizational culture is a key hidden curricular factor and accounts for a large portion of the hidden curriculum. Given the characteristics of clinical environments and the need for inter-professional collaboration, inter-professional and inter-disciplinary cooperation and interaction were mentioned as other important hidden curriculum factors in residency programs. The behavior, habits, and beliefs of residents were frequently observed in the reviewed studies. This indicates the significant impact of these factors on the hidden curriculum of residency programs. Other important factors include leadership and management, hierarchy, professional identity, unplanned teaching and learning, productivity and self-efficacy, and communion. 

**Figure 6:**
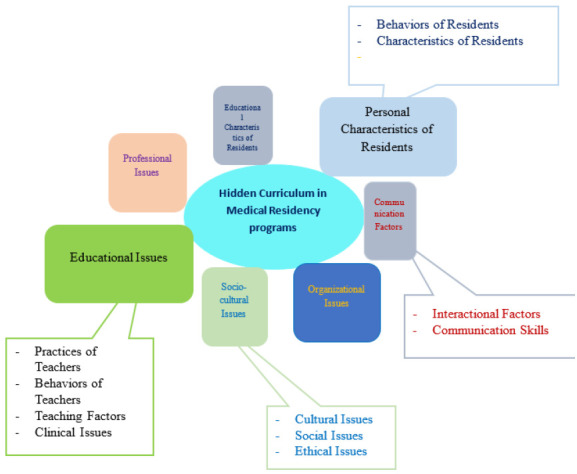
Basic framework of hidden curriculum components and dimensions in the medical residency programs

Once these factors had been divided into subthemes, institutional factors, professional factors, teacher performance, resident behavior, and resident characteristics had the highest weights of codes. This indicates that these subthemes had more effects than other subthemes on the hidden curriculum in residency programs. Also, among the themes, educational factors and personal characteristics of residents involved the highest number of codes and thus the largest weights, while communication factors and educational resident characteristics had the smallest weights. Fig. 6 represents the residency hidden curriculum framework using the extracted themes and subthemes. This framework can be extended by conducting further studies. This initial framework determines the sizes of the themes based on the weights of their codes; in this regard, educational factors have the largest size, while communication factors have the smallest size.

This section discusses the hidden curriculum factors of residency programs extracted from previous studies. Rothlind, et al. (2020) identified family medicine residency themes, including cultural factors, professionalization, and uncertainty. The results of the present study are consistent with Rothlind, et al. (2020) in regard to cultural factors and professionalization ( 66 ). However, the findings of the present study did not emphasize uncertainty. Gupta, et al. (2020) studied psychiatry residency programs in Canada, concluding that inter-discipline and role modeling factors had strong relationships with the hidden curriculum in the creation of professional identity ( 63 ). Herr, et al. (2020) presented a review and proposed role modeling, professionalization, environmental factors, unprofessional communication, isolation, and humiliation to be hidden curricular factors that were experienced by radiology residents ( 68 ). Consistent with the present study, Payne, et al. (2019) and Wild, et al. (2018) identified social factors, role modeling, and professional identity as residency hidden curriculum factors ( 38 , 67 ). As with many other studies, Kelly and Mullan (2018) found that professionalization, organizational culture and structure, sympathy, and communion were the main residency hidden curriculum factors ( 69 ). Lehmann, et al. (2018) presented a comprehensive list of hidden curriculum factors, including role modeling, professionalization, values, norms, culture, curiosity, respect and sympathy, and reflection ( 41 ). Furthermore, hierarchy, professionalization, role modeling, organizational culture, environmental culture, inter-professional communication, inter-discipline communication, personal communication, values, attitudes, beliefs, individual responsibilities and ethics, norms, feedback, unplanned education, and unpredicted education are among hidden curriculum factors of residency programs mentioned in most residency hidden curriculum-related researches (Van den Heuvel, et al. (2017), Garshasbi, et al. (2017), Gupta, et al. (2016), Meiboom, et al. (2015), Martinez and Lehmann (2013), Adkoli, et al (2011), Billings, et al. (2011), Nittur and Kibble (2017) and Beach, et al. (2020)) ( 19 , 29 , 43 , 45 , 47 , 49 , 60 , 64 , 65 ). 

Consistent with the present study, Nothnagle, et al. (2014) and Van-Deven, et al. (2013) mentioned a number of factors, such as professionalization and professional identity, role modeling, professional and organizational culture, organizational rules and structure, and individual and professional values, and emphasized educational factors, including self-oriented learning skills, rethinking, objective setting, teacher-student interaction, clinical educational environment, mentoring, guidance, feedback, and educational support ( 27 , 46 ). 

A number of studies on the hidden curriculum of residency programs mentioned hidden curriculum aspects and factors somewhat different from the present study. Mackin, et al. (2019) identified factors such as vulnerability, privilege, humiliation, hierarchy, guidance and negotiation, positivism, organizational culture and structure, emotional and professional growth, physician-patient interaction, and resident productivity ( 55 ). Esteghamati, et al. (2016) mentioned professionalization, communication skills, and role modeling as well as contextual and cultural factors, hospital and clinical climate and culture, hierarchy, treatment priority over education, multiple physician responsibilities, and clinical priorities as hidden curriculum factors of residency programs. Uscátegui-Daccarett, et al. (2016) identified rules, habits, beliefs, values, context and professionalization, integration, ethics, teacher attitudes, colleague and patient relationships, student-teacher interaction, welfare and academic aspects, and the encouragement of human, art, and cultural activities as hidden curriculum factors ( 73 ). 

Doja, et al. (2018) reported relatively different results in relation to pediatric residency hidden curriculum factors. They suggested authority hierarchy, communication, and circulation in clinical areas as the themes of their framework ( 39 ). Hanson, et al. (2017) mentioned not only the aforementioned common factors, e.g., organizational culture and sympathy, but also social conscientiousness and real-life experiences ( 57 ). Balmer, et al. (2015) proposed role modeling and learning-by-doing as the key hidden curriculum factors from the perspective of residents ( 59 ). 

### Limitations

The present study encountered a number of limitations. First, this work adopted the scoping review methodology. Therefore, the evaluation process was not as strict as a systematic review since a scoping review presents a general perspective of the subject and can identify themes. Hence, the present study is to be exploited to obtain awareness of general trends and as a background for further studies on the subject under discussion. Second, the subject of the study was somewhat unique with limited foreign grey work availability. Thus, the search process did not involve grey work on hidden curriculum of residency programs. The present work attempted to perform a complete search with no missed reference; however, there might have been missed references. 

### Future research suggestions

The adopted scoping review methodology presents a general perspective of hidden curriculum of residency programs, in the sense that it identifies the themes of the subject under study. The present work described the themes and factors of the residency hidden curriculum and proposed a conceptual framework of the residency hidden curriculum. Hence, it can be used as a basis for further and more specialized researches to obtain deeper and context-based insights, extend the conceptual framework of the residency hidden curriculum, and separate the factors based on the residency discipline, to conduct complementary studies by qualitative methodologies, such as ethnography, phenomenology, and grounded theory, using data collection techniques such as interviews and observation are recommended.

## Conclusion

The present study evaluated the hidden curriculum of residency programs. Despite the limited literature, this study identified factors and components and proposed an initial framework for hidden curriculum of residency programs. The results obtained from the review of 43 studies could be exploited by residency education researchers and practitioners to realize residency hidden curriculum factors. This allows for diminishing negative impacts and enhancing positive impacts of the hidden curriculum in residency programs. Indeed, to perform complementary research and to extend the findings, more extensive and deeper studies with qualitative or hybrid methodologies are recommended to further investigate hidden curriculum in different contexts and disciplines. The present study may be exploited as the basis of such research to extract initial questions. The knowledge provided by the present work and complementary studies would be helpful in taking effective and cost-efficient steps toward the design and development of curriculum of residency programs and educational environments in different contexts and disciplines, enhancing the positive, beneficial aspects of hidden curriculum. 

## Acknowledgement

The authors express their sincere gratitude to the librarianship and information technology specialists of the Central Library of Iran University of Medical Sciences who provided access to databases and electronic information resources.

**Conflict of Interest:** None Declared.
